# SecA Localization and SecA-Dependent Secretion Occurs at New Division Septa in Group B Streptococcus

**DOI:** 10.1371/journal.pone.0065832

**Published:** 2013-06-07

**Authors:** Sara Brega, Elise Caliot, Patrick Trieu-Cuot, Shaynoor Dramsi

**Affiliations:** 1 Institut Pasteur, Unité de Biologie des Bactéries Pathogènes à Gram positif, Paris, France; 2 CNRS, ERL 3526, Paris, France; Instituto Butantan, Brazil

## Abstract

Exported proteins of *Streptococcus agalactiae* (GBS), which include proteins localized to the bacterial surface or secreted into the extracellular environment, are key players for commensal and pathogenic interactions in the mammalian host. These proteins are transported across the cytoplasmic membrane via the general SecA secretory pathway and those containing the so-called LPXTG sorting motif are covalently attached to the peptidoglycan by sortase A. How SecA, sortase A, and LPXTG proteins are spatially distributed in GBS is not known. In the close relative *Streptococcus pyogenes*, it was shown that presence of the YSIRKG/S motif (literally YSIRK_X3_Gx_2_S) in the signal peptide (SP) constitutes the targeting information for secretion at the septum. Here, using conventional and deconvolution immunofluorescence analyses, we have studied in GBS strain NEM316 the localization of SecA, SrtA, and the secreted protein Bsp whose signal peptide contains a canonical YSIRKG/S motif (YSLRKykfGlaS). Replacing the SP of Bsp with four other SPs containing or not the YSIRKG/S motif did not alter the localized secretion of Bsp at the equatorial ring. Our results indicate that secretion and cell wall-anchoring machineries are localized at the division septum. Cell wall- anchored proteins displayed polar (PilB, Gbs0791), punctuate (CspA) or uniform distribution (Alp2) on the bacterial surface. *De novo* secretion of Gbs0791 following trypsin treatment indicates that it is secreted at the septum, then redistributed along the lateral sides, and finally accumulated to the poles. We conclude that the ±YSIRK SP rule driving compartimentalized secretion is not true in *S. agalactiae*.

## Introduction

*Streptococcus agalactiae* (also known as Group B Streptococcus, GBS) is an extracellular gram-positive encapsulated bacterium that colonizes the adult gastrointestinal tract and vaginal flora of 15–30% of the healthy women [1]. When transmitted from a ‘carrier’ mother to newborn infants, this opportunistic pathogen can provoke invasive neonatal diseases, including sepsis, pneumonia, and meningitis [2]. Besides neonatal infections, *S. agalactiae* is an emerging pathogen in the elderly and in individuals suffering from underlying diseases [3]. Exported proteins, which include proteins localized to the bacterial surface or secreted into the extracellular environment, are major determinants of virulence in *S. agalactiae* as they play key roles in colonization of host tissues and control of immune responses [4]. Most bacterial proteins that translocate across the cytoplasmic membrane possess a N-terminal signal peptide and are exported to extracellular compartments via the highly conserved general secretory SecA-YEG pathway. These secreted proteins are either released into the extracellular milieu or anchored to the cell wall by the housekeeping class A sortase if they possess a C-terminal LPXTG cell wall sorting signal consisting of an LPXTG-like motif followed by a hydrophobic domain and a positively charged tail [5]. SrtA cleaves the LPXTG motif and links the carboxyl-group of the threonine to the peptidoglycan precursor lipid II [6]. The protein-lipid II-linked product is then incorporated into the mature peptidoglycan (PG) *via* the cell wall biosynthesis machinery. *In silico* analysis of *S. agalactiae* NEM316 genome predicts the existence 35 LPXTG proteins [7] thought to be covalently attached to the cell wall by a unique SrtA [8].

In the close relative *Streptococcus pyogenes*, Rosch and Caparon showed that the SecA motor and secreted proteins specifically localize to a microdomain named ExPortal which is distal to the poles [9]. This finding was subsequently challenged by Carlsson *et al.* who showed a uniform distribution of SecA in the membrane of *S. pyogenes*. Remarkably, Carlsson et al. also found that the signal peptide of M protein, which contains an YSIRK motif (SP_+YSIRK_), directs secretion to the division septum, while that of protein F (PrtF, also known as SfbI) lacking this motif (SP_−YSIRK_) targets the old pole [10]. Recently, Raz *et al.* confirmed the differential localization of M and PrtF/SfbI proteins in *S. pyogenes* and further provided a dynamic 3D view of protein localization during cell cycle [11]. The M protein is rapidly anchored at the septum, simultaneously at the mother and daughter septa. In contrast the SP_−YSIRK_ SfbI protein accumulates gradually on peripheral peptidoglycan resulting in a polar distribution. Protein localization is not perturbed in a SrtA mutant, suggesting that signal sequence directs localized secretion before cell wall anchoring [11]. In contrast, impairment of septum assembly results in marked reduction in the amount of M protein, but not of Sfbl. A functional divisome is thus critical in the anchoring of SP_+YSIRK_-type proteins and may control their expression/stability [11].

The signal peptide-dependent protein localization rule is also true in *Staphylococcus aureus*. SP_+YSIRK_ CWA proteins (protein A, ClfA, SdrC, SdrD, and FnbpB) were shown to be distributed in a ring-like manner near the vicinity of the cell division site, whereas SP_−YSIRK_ proteins (SasA, SasD, SasF, and SasK) were found at discrete peripheral loci on the bacterial envelope [12]. Recently, generation of a mCherry reporter system to investigate the spatial localization of surface proteins in live *S. aureus* further confirmed that SP_+YSIRK_ and SP_−YSIRK_ are necessary and sufficient to drive localization of CWA proteins either to the equator/septum or pole, respectively [13]. Interestingly, treatment with sub-lethal concentrations of penicillin shifted the localization of the non-YSIRK mCherry hybrids from the peripheral cell wall to the septum in a SrtA-dependent manner. This relocalization may be due to increased amounts of lipid II precursors at the septum [13].

In the present work, we investigated the subcellular localization of SecA, SrtA, two secreted proteins (Bsp and CAMP), and four cell wall-anchored proteins (Alp2, PilB, CspA and Gbs0791) in the coccoid *S. agalactiae* using immunofluorescence microscopy. Our main finding is that SecA and SrtA are both localized at the division septum suggesting that SecA-dependent secretion and SrtA-dependent cell wall anchoring are spatially coupled in *S. agalactiae*. Thus, the polar distribution of some LPXTG proteins (e.g. PilB and Gbs0791) likely reflects a post-secretion process dependent on SrtA.

## Results

### SecA localizes at equatorial rings and division septa in exponentially growing GBS

**Figure 1:**
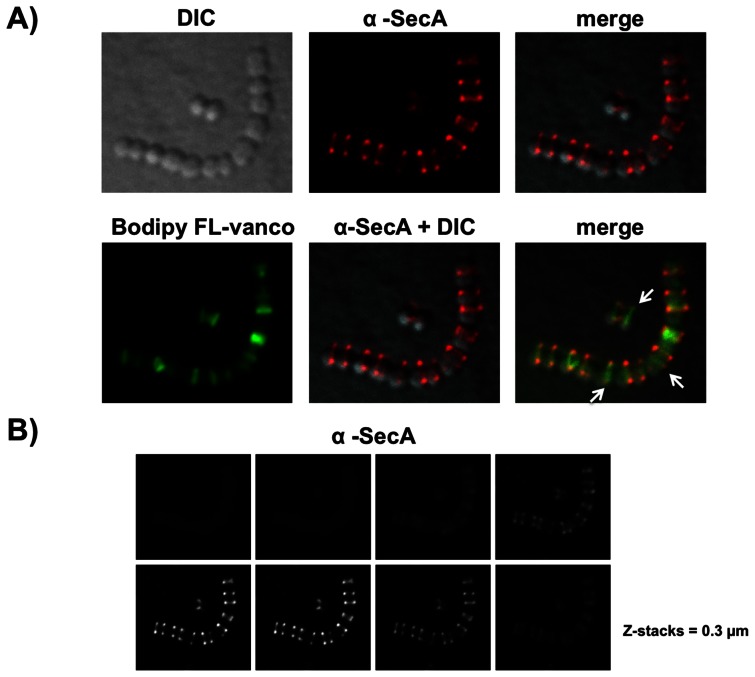
Distribution of SecA in *S.* *agalactiae* NEM316. Bacteria grown overnight in 10 ml of TH (OD_600_≈2) were diluted to get an initial OD_600_ of 0.05 (1/40 dilution) and grown at 37°C until OD_600_ reached 0.5 and re-diluted again in TH (1/10) until reaching an OD_600_ of 0.5 and diluted again (1/10) before final collection at mid-exponential phase (OD_600_ of 0.3) to get a homogenous population of exponentially growing cells. Bacteria were pretreated with lysozyme (1 mg/mL final concentration) for 15 min at 37°C and then permeabilized with PBS-Triton X-100 (0.4%) for 5 min at RT, washed twice with PBS and then fixed with PBS containing 3% paraformaldehyde for 15 min at RT. (A) Differential interference contrast (DIC) and immunofluorescence microscopy (IFM) of bacteria harvested in mid-exponential phase and visualized with rabbit anti-SecA pAb (red) or fluorescent vancomycin (green) plus rabbit anti-SecA pAb (red). White arrows in the last panel indicate potential constriction septa. Image representative of at least 800 GBS chains analyzed (B) Deconvolution images of sequential z-sections (0.3 µm) of NEM316 cells labeled with rabbit anti-SecA pAb presented as maximum intensity projections.

**Figure 2:**
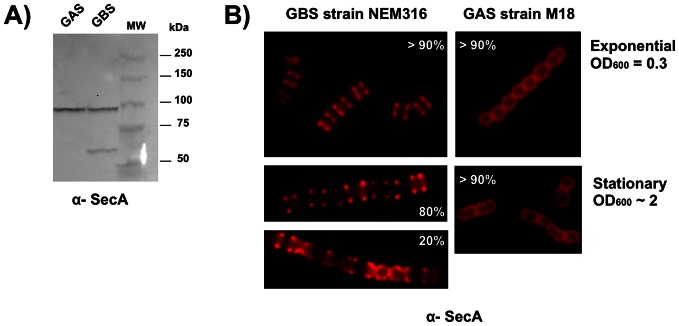
Distribution of SecA at the surface of *S.* *agalactiae* (GBS NEM316) and *S. pyogenes* (GAS M18). (A) Western blot image showing that the polyclonal antibody directed against *E. coli* SecA recognizes a band of approximately 90 kDa in both GAS M18 and GBS NEM316 strains. (B) Conventional immunofluorescence microscopy showing the differential distribution of SecA at the surface of GBS NEM316 versus GAS strain M18 collected in exponential and stationary phase of growth. Heterogeneity of SecA distribution was quantified by eye following analysis of randomly selected fields.

Despite its importance to bacterial physiology and virulence, the subcellular localization of SecA of *S. agalactiae* has never been reported. We determined the localization of SecA in exponentially growing cells using a polyclonal rabbit antibody raised against SecA of *E. coli*. We first checked the specificity of this antibody and showed that it reacts with a single band of the expected size of about 90 kDa in a Western blot analysis of whole cell extracts of *S. agalactiae* strain NEM316 and *E. coli* strain DH5α as a positive control (Fig. S1). Immunofluorescence microscopy analysis revealed a defined localization of SecA at equatorial ring, i.e. a zone of active peptidoglycan synthesis, in growing streptococcal chains (Fig. 1A). Quantitative image analysis of 15 different fields at lower magnification (e.g. Fig. S2) showed that amongst 880 bacteria counted, 797 formed a chain (>2 cocci) with the typical SecA labeling as shown in Fig. 1 (90%), 81 single cocci displayed a uniform labeling (9%, Fig. S2), and only 2 (0.2%) were not labeled. Ovococcal bacteria, like streptococci, divide along the perpendicular axis of a chain and rely on the activity of two finely coordinated peptidoglycan machineries at the mid-cell that are dedicated either to cell elongation or septation [14]. Fluorescent-vancomycin, which binds to peptidoglycan (PG) precursor [15], is a useful tool to visualize novel sites of PG synthesis on streptococcal chains [16]. Double fluorescence labeling experiment using both anti-SecA antibody and fluorescent vancomycin indicates that SecA and vancomycin mostly co-localizes at septal sites (Fig. 1A). Of note, a few septal sites that potentially correspond to constriction sites were not labeled with SecA (white arrows in Fig. 1, last panel). Deconvolution microscopy confirmed the localization of SecA at equatorial septa in GBS strain NEM316 (Fig. 1B). In contrast, we found that SecA was uniformly distributed on the bacterial surface of GAS strains (Fig. 2) as previously published [10].

### Extracellular proteins are found at the division septa of *S. agalactiae*

**Figure 3:**
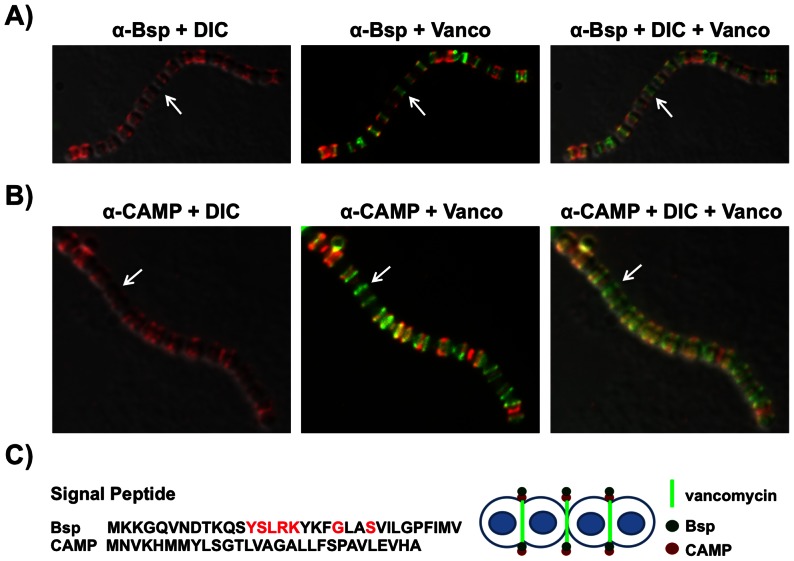
Subcellular localization of Bsp and CAMP factor in *S.* *agalactiae* NEM316. (A, B) IFM images of bacteria harvested in exponential phase (OD_600_ = 0.3) and labeled with specific antibodies directed against Bsp (A) or CAMP factor (B) revealed with AlexaFluor 594- fluorescent secondary antibody (red). Outline of the cells was visualized by DIC and active zone of peptidoglycan synthesis with fluorescent vancomycin (green). (C) Signal peptides of Bsp and CAMP proteins. The amino acids constituting the YSIRK motif are highlighted in red. Schematic localization of vancomycin, Bsp, and CAMP factor at non-constricting septa.

Bsp (group B secreted protein) is a highly conserved extracellular protein of 65 kDa [17] encoded by *gbs1420* in strain NEM316 genome [7]. This protein exhibits limited similarity (∼25%) to the murein hydrolase lysostaphin and possesses four SH3 domains and an atypical LPXTG signature lacking the downstream hydrophobic sequence. In line with this observation, Bsp protein is found almost exclusively in the culture supernatant [17]. GBS also secretes another well-characterized 25 kDa pore-forming toxin known as CAMP factor [18]–[20]. To investigate the subcellular distribution of Bsp and CAMP proteins, we made use of specific polyclonal antibodies against Bsp and CAMP that were previously used [21], [22]. Using deconvolution immunofluorescence microscopy, we showed that Bsp and CAMP proteins co-localize with green fluorescent vancomycin at septal sites in strain NEM316 (Fig. 3). As for SecA labeling, a few division sites that may correspond to constrictions sites were not labeled with Bsp or CAMP (white arrows).

### Localization of Bsp to the septum does not depend on a YSIRK signal peptide

The signal peptide dependent localization rule defined in GAS [10], [13] and *S. aureus* [12] specifies that a signal peptide containing the YSIRK/GS motif directs secretion close to the cell division site (septum), whereas non-YSIRK signal peptide directs proteins near the poles. However, this rule is apparently not observed in GBS (Fig. 3) as Bsp contains a typical YSIRK signal peptide but not the CAMP protein (Fig. 3C).

**Figure 4:**
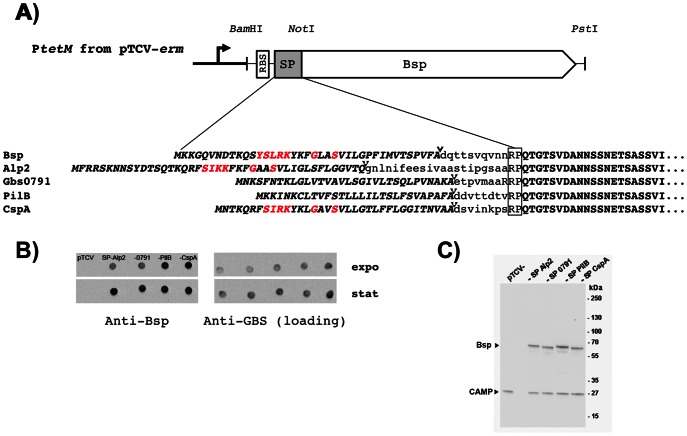
Expression in GBS of Bsp recombinant proteins with structurally unrelated signal peptides. (A) *Bam*HI-*Not*I PCR fragments carrying the ribosome binding site (RBS) and the signal peptides (SP) of 5 SecA-dependent substrates (Bsp, Alp2, Gbs0791, PilB, and CspA) were fused in frame with a *Not*I-*Pst*I PCR fragment coding the Bsp protein devoid of its signal peptide (Table S1). The resulting *Bam*HI-*Pst*I fragments were cloned downstream the constitutive P*tetM* promoter from the low-copy-number pTCV*-erm*. The SP and Bsp sequences are indicated in upper-bold italic characters and upper-bold characters, respectively. The boxed RP motif in all proteins corresponded to the translation of the two internal codons of the *Not*I restriction site (CGGCCG). All but one SP were predicted with SignalP 4.1 (www.cbs.dtu.dk/services/SignalP/) whereas the remaining (Alp2) was predicted with PrediSi (www.predisi.de). The AA residues in the SP thought to direct localized secretion at the bacterial surface are indicated in red characters. Arrowheads indicate the predicted site of cleavage of the various SP. (B) Analysis of surface display of Bsp recombinant proteins in a Δ*bsp* mutant strain by immunoblotting. Whole bacterial cells harvested in exponential (OD_600_ 0.3) or stationary (OD_600_ 1.2) phases were washed, resuspended in phosphate buffer saline to similar density and spotted on nitrocellulose. Membranes were hybridized with specific anti-Bsp antibodies or with anti-GBS pAb (loading control). (C) Western blotting analysis of culture supernatants. Proteins were separated on 4–12% gradient Tris-acetate Criterion XT SDS-PAGE gel, then transferred onto a nitrocellulose membrane, and detected by immunoblotting with specific anti-Bsp and anti-CAMP antibodies. In (B) and (C), the Δ*bsp* mutant strain harboring pTCV-*erm* (negative control) or pTCV-*erm* directing synthesis of recombinant Bsp proteins associated with Alp2, Gbs0791, PilB, and CspA signal peptides were used.

**Figure 5:**
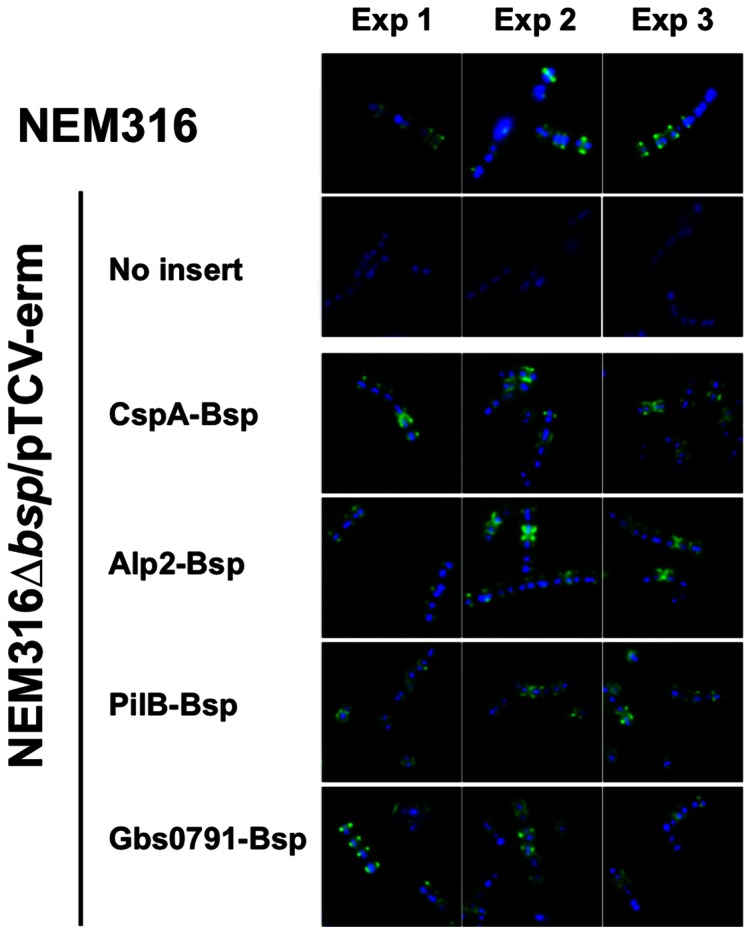
Surface distribution of Bsp in *S.* *agalactiae* NEM316 and derivatives. Bacteria were harvested in exponential phase (OD_600_ = 0.3) and labeled with DAPI (blue) plus rabbit anti-Bsp pAb (green). The bacterial strains studied were: NEM316 (WT) (positive control); NEM316Δ*bsp*/pTCV-erm without insert (negative control); NEM316Δ*bsp*/pTCV-*erm* expressing recombinant Bsp with the signal peptide of CspA, Alp2, PilB, or Gbs0791. Data are representative of three independent experiments.

To further test this localization rule in GBS, we decided to swap the signal peptide of Bsp with those of four cell wall-anchored proteins, two containing the YSIRK motif (Alp2 and CspA) and two without it (PilB and Gbs0791), and the chimeric genes were expressed in the low-copy number plasmid pTCV-*erm* under the control of the constitutive Tn*916 tetM* promoter (P*tetM*). A schematic representation of theses constructs is shown in Fig. 4A. We first showed that the various signal peptides allowed the secretion of Bsp, as shown by dot blot on whole bacteria using exponential and stationary growing bacteria (Fig. 4B). Western blotting of supernatant protein extracts revealed that the four different constructs directed synthesis of a specific band of about 60 kDa corresponding to Bsp, while the control vector did not (Fig. 4C). This result demonstrates that YSIRK- and non-YSIRK containing signal peptides enable the secretion of Bsp at similar levels (Fig. 4C). We next examined by immunofluorescence microscopy the localization of these four secreted Bsp differing by their signal peptides (Fig. 5). In exponentially growing bacteria, all Bsp isoforms were mainly localized at the septum and thus, we concluded that localization of a secreted protein in *S. agalactiae* is independent of the YSIRK motif.

### Subcellular localization of SrtA- and SrtA- substrates in *S. agalactiae*

**Figure 6:**
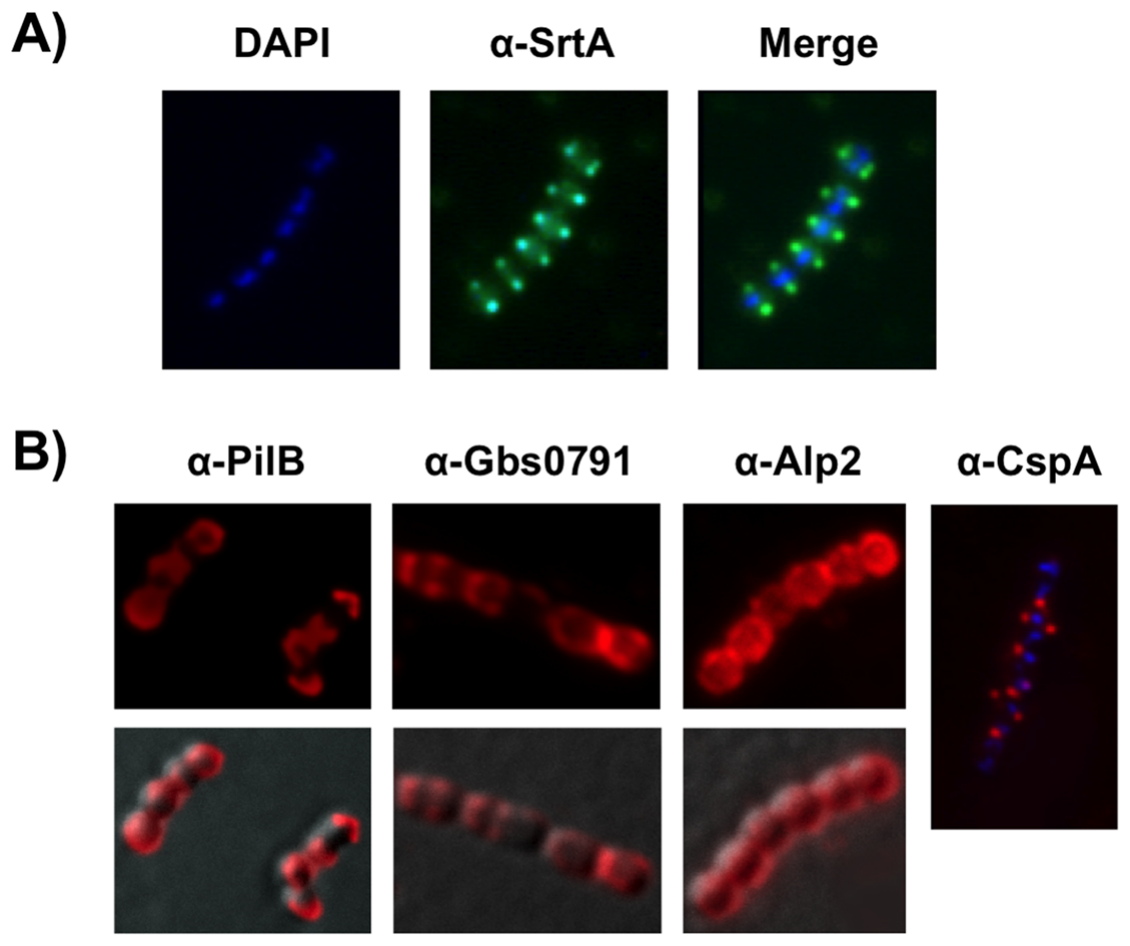
Surface localization of SrtA and unrelated cell wall-anchored proteins in *S.* *agalactiae* NEM316. (A, B) Bacteria harvested in exponential phase (OD_600_ = 0.3) were labeled with (A) DAPI (blue) or guinea pig anti-SrtA pAb (green) and (B) with rabbit pAb against PilB, Gbs0791, Alp2, or CspA (red).

We next aimed at characterizing the subcellular distribution of the cell wall anchoring enzyme SrtA and of some SrtA-substrates for which specific antibodies were available. We first checked the specificity of the antibody raised against GBS SrtA. Western blotting showed a band at approximately 30 kDa (SrtA ca MW 27,503.7) in the total protein extracts of NEM316 strain that was absent in the isogenic Δ*srtA* mutant (not shown). Immunofluorescence analysis showed that SrtA, like SecA, localizes at equatorial rings and division septa in exponentially growing GBS (Fig. 6A). However, it is worth to mention that SrtA showed a more diverse distribution than SecA (Fig. S2). We next analyzed the subcellular distribution of four LPXTG-containing proteins that are anchored to the cell wall by the SrtA sortase (Fig. 6B). These were PilB, the major pilin of the PI-2A pilus [23]; Gbs0791, a highly conserved protein of unknown function (our unpublished data); Alp2, a major surface protein [24]; and CspA, a surface serine protease that cleaves human fibrinogen [25] and CXC chemokines [26]. Note that PilB and Gbs0791 do not contain the YSIRK motif in their signal peptides whereas Alp2 and CspA do possess the YSIRK motif (Fig. 4). Surprisingly, although the septal localization of SecA and SrtA machineries in *S. agalactiae* strongly suggest that secretion of cell wall-anchored proteins occurs at the septum, we observed that these cell wall-anchored proteins displayed various patterns: polar (PilB, Gbs0791), punctuate (CspA) or uniform distribution (Alp2) on the bacterial surface (Fig. 6B). We interpret these results as indicating that these proteins were dynamically redistributed on the bacterial surface after their secretion.

### *De novo* secretion of Gbs0791 occurs at the septum

**Figure 7:**
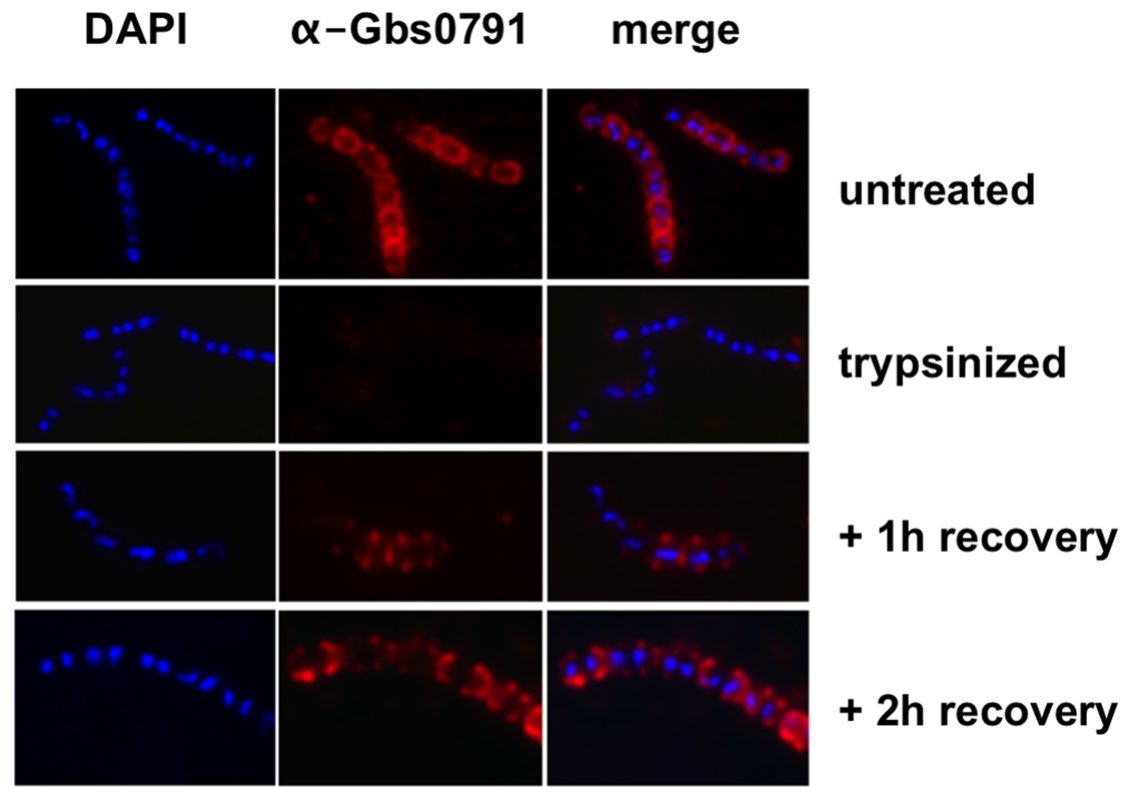
Surface distribution of newly synthesized Gbs0791 in *S.* *agalactiae* NEM316. Bacteria were harvested in exponential phase (OD_600_ = 0.3), incubated for 1 h with trypsin (0.1 mg/ml), washed twice in TH broth, and incubated at 37°C for 1 h and 2 h. Untreated or trypsinized cells after various recovery times were labeled with DAPI (blue) or rabbit anti-Gbs0791 pAb (red).

The redistribution of a polar protein on the GBS surface was further studied as follows. Since PilB containing pili are highly resistant to trypsin and proteinase K treatments (data not shown), we chose to analyze the site of Gbs0791 *de novo* secretion after shaving the bacterial surface with trypsin as previously described [10]. Immunofluorescence analysis showed that Gbs0791 was rapidly and efficiently removed by trypsin from the bacterial surface. We observed that this protein re-appeared at septal sites after 1 h of regeneration, was later redistributed laterally to recover the entire bacterial surface after 2 h of regeneration, and finally displayed a preferential polar localization in stationary phase bacteria (Fig. 7).

### Protein localization is strongly altered in a non-capsulated mutant

**Figure 8:**
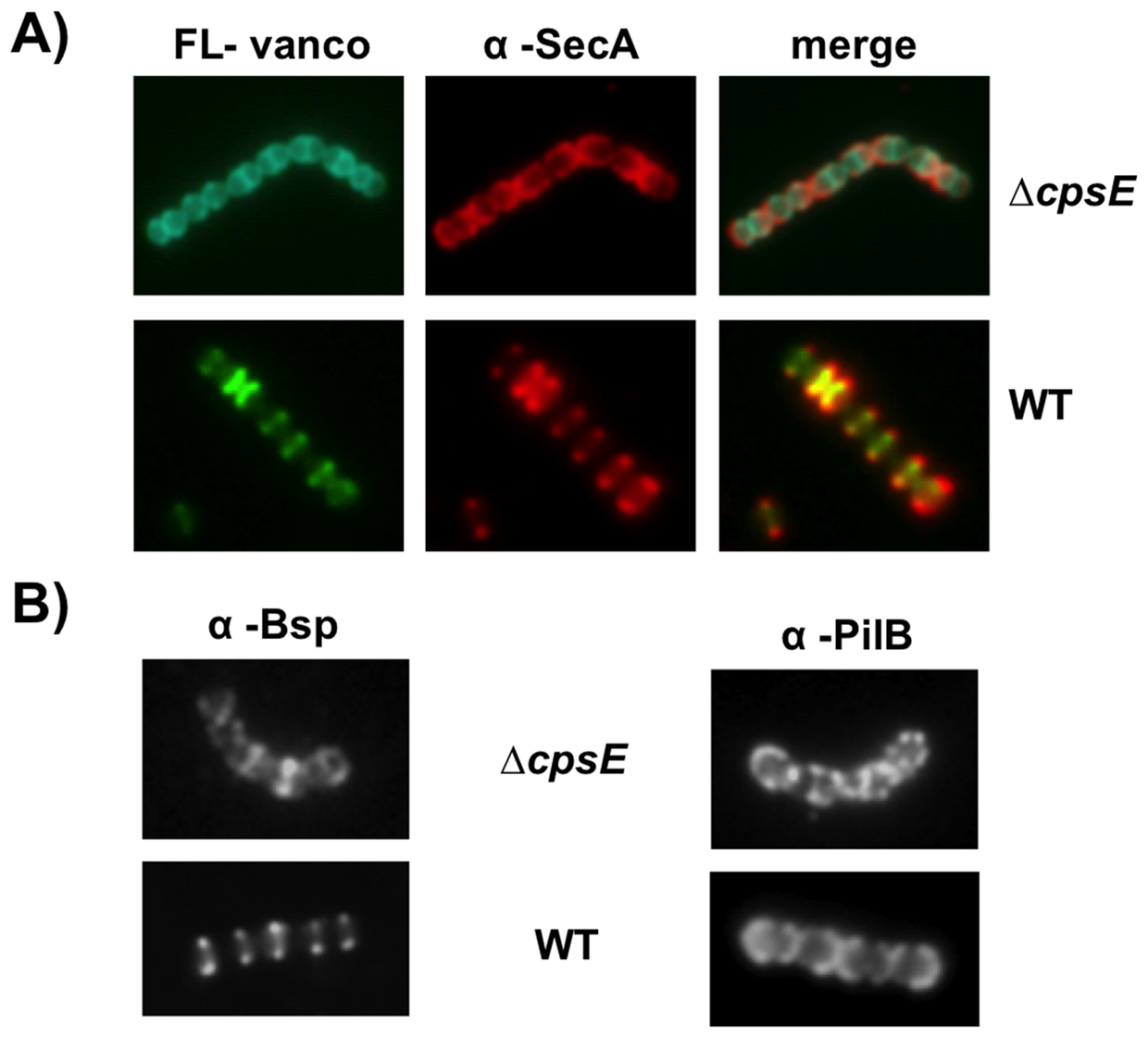
Conventional immunofluorescence microscopy showing localization of SecA in the non-capsulated mutant Δ*cpsE* compared to the parental WT NEM316. (A) Immunofluorescence microscopy of bacteria harvested in mid-exponential phase and visualized with fluorescent vancomycin (green) or plus rabbit anti-SecA pAb (red). Note that SecA is more concentrated in the constricting septa and its neighboring region in a pattern very similar to that reported for the non-capsulated strain of *S. pneumoniae* [31]. (B) Immunofluorescence of bacteria harvested in mid-exponential phase and visualized with rabbit pAb against Bsp and PilB.

The capsule of *S. agalactiae* is an extracellular polysaccharide that plays a key role in virulence [27]. Immunofluorescence analysis of SecA distribution in the non-capsulated Δ*cpsE* mutant showed a more diffuse labeling compared to the defined labeling in the parental strain NEM316 (Fig. 8A). Similarly, localization of the secreted Bsp and the cell wall-anchored PilB is strongly altered in the non-capsulated mutant as compared to the parental NEM316 strain (Fig. 8B).

## Discussion

In this report, we describe the first subcellular localization of endogenous unrelated secreted proteins in the coccoid *Streptococcus agalactiae* (GBS), as determined by immunofluorescence microscopy using specific antibodies. These were the general motor ATPase SecA of the general secretory pathway, two known secreted proteins Bsp and CAMP, the cell wall anchoring enzyme SrtA, and four SrtA-substrates (also known as LPXTG-containing proteins). Our main finding is that SecA-dependent secretion and SrtA anchoring machineries are spatially coupled and localized at the division septum in exponentially growing culture of *S. agalactiae*, collected after successive dilutions, to get a homogeneous population of individual cells (OD_600_ of 0.3).

We found that Bsp and CAMP, two abundantly secreted proteins, were also localized at the division septa in exponentially growing bacteria. Sequence analysis revealed that the Bsp signal peptides displayed a canonical YSIRK motif whereas the CAMP signal peptide did not, which in turn suggested that the YSIRK signal peptide-dependent localization rule did not apply to *S. agalactiae*. This rule, first proposed in *S. pyogenes* [10] and later confirmed in *S. aureus* [12], stipulates that YSIRK-containing signal peptide drives the localization of a reporter protein to the septum whereas non-YSIRK signal peptide targets the reporter protein to the pole [13]. Intriguingly, mutations in the conserved YSIRK motif did not change the localization pattern of the corresponding proteins [10], [12], suggesting that this motif is not *per se* the localization signal but rather a “tag” for a class of signal peptides with specific properties. Of note, deletion of SP_+YSIRK_ in protein A from *S. aureus* reduced the amount of anchored polypeptide [28]. Indeed, the SP_+YSIRK_-mCherry chimera is more highly expressed than the SP_−YSIRK_-mCherry chimera [13].

In this work, the GBS protein Bsp was chosen as a secretion reporter protein and we constructed a set of variants by replacing its native SP with that of four *S. agalactiae* cell wall-anchored proteins, two containing (Alp2 and CspA) and two lacking (PilB and Gbs0791) the YSIRK motif. To avoid artifacts due to interferences with the native protein and/or differential expression levels, all recombinants proteins were similarly expressed from the same vector into NEM316:Δ*bsp* mutant strain. We first showed that all tested SP allowed the secretion of Bsp in the culture supernatant at approximately the same level (Fig. 4). Importantly, as shown in Fig. 5, the localization pattern is very similar to that of the native Bsp in WT strain NEM316, with a majority of bacteria displaying a septal localization of Bsp that appears to be independent of the signal peptide directing its secretion. It is worth noting that increasing the amount of Bsp on the bacterial surface through overexpression was associated with the presence of a significant increase amount of protein to the lateral sites, but not with the redistribution of the protein to the poles (data not shown).

Very few localization studies have been performed in *S. agalactiae*. In particular, the highly conserved protein Sip (NEM316 Gbs0031), the group B surface immunogenic protein, has been found at septal and polar regions by immunogold electron microscopy [29]. The signal peptide of Sip lacks the YSIRK motif but the mature secreted protein contains a LysM domain that might mediate binding to the peptidoglycan. Sip has been localized both at the cell surface and secreted in the culture supernatant of all GBS strains tested [29]. How can we explain the polar localization of Sip? In streptococci, which divide in parallel plane along the short axis of the bacterial chain, elongation occurs faster than division and therefore future septa of daughter cell are visible in a bacterial chain before cell separation. Therefore, polar distribution of a protein could result either from an active targeting to the poles or a passive targeting occurring when the septum turns into new pole following bacterial division. From our results on the localization of SecA and other secreted proteins, we hypothesize that secretion occurs at the septum and protein accumulation at the poles is the result of long-lived proteins.

In related streptococci, different localization patterns have been described for both SecA and sortase A (for a recent review, see [37]). In *S. pyogenes*, Rosch *et al.* proposed that SecA colocalizes with sortase A in a single membranal microdomain termed ExPortal [9], [30]. This domain is enriched in anionic lipids and thus can be visualized using a dye such as N-nonyl-acridine orange NAO. However, another study, revealed that SecA is distributed uniformly [10], a result that we confirmed using our anti-SecA antibody on GAS M18 and M49 strains (Fig. 2 and data not shown). Recently, *S. pneumoniae* SecA and SecY were localized in the mid-cell region, whereas the sortase A was found over the surface of cells without discernable pattern [31]. An ExPortal–like anionic lipid microdomain similar to that described previously in GAS [32] was detected in GBS NEM316, but it is clearly not associated with SecA equatorial localization (Fig. S3). This observation rules out the model of ExPortal in *S. agalactiae*. Consistently, we similarly showed that SrtA is localized mainly at the septum reinforcing the idea of a multi-protein complex coupling protein secretion, cell wall- anchoring and peptidoglycan synthesis at the division site. Interestingly, SecA, Bsp, and CAMP labeling seem to disappear from constricting septa (Fig. 1 and 3), an observation that needs further investigation using video-microscopy. SrtA was found mostly at the septum, but also as discrete foci as described for SecA in *Bacillus subtilis* [33] or randomly over the entire surface like in *S. pneumoniae* [31]. Localization of four GBS sortase A-substrates revealed very different subcellular distributions that can be defined as uniform (Alp2), polar (PilB and Gbs0791), or punctuated (CspA), as compared to the septal distribution of SrtA. As LPXTG containing proteins are covalently linked to the cell wall precursor lipid II prior to being incorporated into the mature peptidoglycan [6], one can expect a spatial proximity of the secretion machinery (SecA) with the anchoring (SrtA) and peptidoglycan factories. Streptococci which are elongated ellipsoid organisms called ovococci [14] possess two cell wall synthesis systems. One is associated with septum formation where PG is synthesized at the cell division creating the septal wall required for formation of the new poles; the other is a multi-protein complex required for the slight longitudinal peripheral elongation of their sidewalls prior to division. Indeed, fluorescent vancomycin, which is used to label new peptidoglycan incorporation sites revealed both septal and, to a lesser extent, sidewall localizations (Fig. 3). These observations support the idea that LPXTG proteins are secreted by the SecA machinery at the septum and then immediately anchored to the peptidoglycan precursor by SrtA in *S. agalactiae*. In line with this model, *de novo* synthesis of the LPXTG protein Gbs0791 after trypsin digestion revealed that this protein is secreted at the septum and that polar distribution most probably results from a post-secretion process. We speculate that the different localizations of LPXTG proteins at the bacterial surface likely depend upon several factors: anchoring efficiency which itself depends on protein synthesis levels and stability, protein association with the membrane, protein diffusion rate in the lipid layer, but can also depend on the activity of other LPXTGase as those identified in both *S. pyogenes* and *S. aureus* [34], [35]. We hypothesize that polar localization of highly stable LPXTG proteins, like for example PilB or Srr1 [36], could be the result of protein secretion and anchoring at the septum, followed by redistribution through septal and peripheral peptidoglycan synthesis [14] and accumulation to the poles at later stages.

Surprisingly, the GBS capsular polysaccharide was found to play an important role for the proper spatial localization of surface proteins.

In conclusion, this study constitutes the first report showing the spatial localization of surface proteins in the gram-positive pathogen *Streptococcus agalactiae*. We provide evidence that secretion and cell wall anchoring machineries are localized at the division septum and that the YSIRK- signal peptide-dependent localization rule did not apply to *S. agalactiae*. Our results contrast with those reported previously in the close relative species of *S. pyogenes* but the distribution pattern of SecA in the non-capsulated strains of GBS and *S. pneumoniae* are similar. Thus, we conclude that the localization of surface proteins in a given bacteria is highly specific and probably depends on the organization of other cell surface components such as capsule, extracellular polysaccharide, or lipoteichoic acids.

## Experimental Procedures

### Bacterial strains and growth conditions

*S. agalactiae* NEM316 belongs to the capsular serotype III (ST-23). The complete genome sequence of this strain has been determined [7] and is accessible (NCBI RefSeq NC_004368.1). The uncapsulated *S. agalactiae* NEM316 mutant was described previously and referred to as *cpsD* mutant [27], [38]. However, since a change in the nomenclature of capsular genes, *cpsD* was renamed *cpsE* [39]. *Escherichia coli* DH5α (Gibco-BRL) was used for cloning experiments. *S. agalactiae* (GBS) and *S. pyogenes* (GAS) were cultured in Todd-Hewitt (TH) broth or agar (Difco Laboratories, Detroit, MI) and *E. coli* in Luria-Bertani (LB) medium. *Streptococcus agalactiae* (GBS) and *S. pyogenes* (GAS) strains were cultured in Todd-Hewitt (TH) broth in standing filled tubes without agitation. Erythromycin was used at the following concentrations in µg/mL: for *E. coli*, 150 and for GBS, 10. All incubations were performed at 37°C.

### General DNA techniques

Genomic DNA from *S. agalactiae* was prepared using the DNeasy Blood and Tissue kit (Qiagen). Analytical PCR used standard Taq polymerase (Invitrogen, Life technologies) and preparative PCR for cloning and sequencing was carried out with a high fidelity Phusion polymerase (Finnzymes). Plasmids for overexpression (pTCV backbone) were constructed by standard cloning procedures [40] and all inserts were entirely sequenced to exclude mutations (GATC). All PCR primers used in this study (Bsp translational fusions and histidine-tagged fusion proteins) are listed in Table S1.

### Rabbit and guinea pig polyclonal antibodies

The sequences encoding CspA (residues 36 to 564 with a NH2-terminal 6×His-tag) and Gbs0791 (residues 33 to 361 with a COOH-terminal 6×His-tag) were cloned in pET28 whereas that encoding SrtA (residues 47 to 237 with a COOH-terminal 6×His-tag) was cloned in pET26b. The corresponding histidine-tagged fusion proteins were produced in *E. coli* BL21(λDE3) and purified on nickel-nitrilotriacetic acid (Ni-NTA) agarose as described (www.qiagen.com). Rabbit polyclonal antibodies (pAb) against CpsA and Gbs0791 were obtained from Covalab (www.covalab.com). Guinea pig polyclonal antibodies against SrtA were from Eurogentec (www.eurogentec.com). Rabbit antisera against the CAMP factor (Gbs2000) [23], the PilB pilin (Gbs1477) [24], and the secreted protein Bsp (Gbs1420) [22], were from our antibody collection. Rabbit polyclonal anti-Alp2 antibodies were kindly provided by Gunnar Lindahl (University of Lund, Sweden). Rabbit pAb directed against *E. coli* SecA were purchased from IMBB (www.imbb.forth.gr/groups/minotech-new/antibodies.html; ref 711-1). For double-labelling experiments, mice polyclonal anti-PilB antibodies were obtained at the Institut Pasteur core facilities as described [24].

### Immunofluorescence analyses

*S. agalactiae* NEM316 grown overnight in 10 ml of TH (OD_600_≈2) was diluted to get an initial OD_600_ of 0.05 (1/40 dilution) and grown at 37°C until OD_600_ reached 0.5 and re-diluted again in TH (1/10) until reaching an OD_600_ of 0.5 and diluted again (1/10) before final collection at mid-exponential phase (OD_600_ of 0.3) to get a majority of exponentially growing bacteria. Bacteria were washed twice in phosphate buffered saline (PBS) before fixation in PBS containing 3% paraformaldehyde for 15 min at RT. For SecA detection, bacteria were pretreated with lysozyme (1 mg/mL final concentration) for 15 min at 37°C and then permeabilized with PBS-Triton X-100 (0.4%) for 5 min at RT, washed twice with PBS and then fixed. Fixed bacteria were washed twice with PBS and incubated for 45 min with rabbit or guinea pig primary antibodies diluted in PBS-BSA 0.5% at the following dilutions: Alp2 (1/1000), Bsp (1/200), PilB (1/300), Gbs0791 (1/100), CAMP (1/50), SecA (1/200) and SrtA (1/100). The specificity of Alp2 and PilB antisera has been tested previously [8], [23]. After three washings with PBS, samples were incubated for 30 min with secondary AlexaFluor 488- or AlexaFluor 594- conjugated goat anti-rabbit immunoglobulin (1/300 dilution; Molecular Probes, Invitrogen). Bodipy FL-vancomycin was purchased from Molecular Probes and used at 3 µg/mL for 20 min at RT. Coverslips were mounted with 4 µl of Vectashield mounting medium containing DAPI (Vector Laboratories, Inc). Microscopic observations were done on a Nikon Eclipse E600 and images acquired with a Nikon Digital Camera DXM1200F. Immunofluorescence experiments for SecA and Bsp have been performed at least 20 times independently and images have been analyzed for about 800 bacteria appearing mostly in chains. Immunolocalization of SrtA, PilB, Alp2, and CAMP were performed at least 4 times independently and images are representative of about 100 bacteria.

### Imaging and image processing

Image acquisitions of z-series in wide field fluorescence was performed at the Imagopole on Zeiss Axiovert 200M epifluorescence microscope connected to a CCD camera. Images were acquired with an apochromat 100× (numerical aperture NA 1.4) objective lens coupled to a Piezo system (Princeton Instruments) and processed with Metamorph Software, version 6.1. Stacks of 10 images acquired every 300 nm in the z-axis were deconvolved with the Metamorph Deconvolution module. Maximum projection of the deconvolved stacks of serial optical sections were generated by selecting the highest intensity values along the z-axis and displaying them as an XY.

### Immunoblotting

For analysis of SecA expression, total protein extracts were loaded on SDS-PAGE and transferred to nitrocellulose membrane. SecA was detected using rabbit-specific pAb and Horseradish peroxidase (HRP)-coupled goat anti-rabbit secondary antibody (Zymed). Detection was performed with the Western pico chemiluminescence kit (Thermo Scientific).

## Supporting Information

Figure S1 **Western blotting showing that the polyclonal antibody directed against SecA of** ***E. coli*** **recognizes a band of approximately 90 kDa in GBS despite a low level of identity at the amino acid level.** Western blot showing the specificity of Alp2 antiserum on the cell wall extracts from WT NEM316 and two transposon mutants inserted at two independent sites in *gbs0470* (*alp2*) (M1: NEM3695 and M2: NEM3694).
(TIF)

Figure S2 **Low magnification image of SecA and SrtA labeling in GBS strain NEM316.** Single cocci displaying a uniform labeling are indicated with white arrows.
(TIF)

Figure S3 **An ExPortal-like domain enriched in anionic lipids can be visualized using the NAO (10-N-nonyl-acridine orange 1 µM) dye in GBS strain NEM316.**
(TIF)

Table S1 List of oligonucleotides used in this study.
(DOCX)
