# Supplementary figures and images for: SecA Localization and SecA-Dependent Secretion Occurs at New Division Septa in Group B Streptococcus
Source: PLoS One. 2013 Jun 7;8(6):e65832. doi: 10.1371/journal.pone.0065832 (PMC3676364; doi:10.1371/journal.pone.0065832)

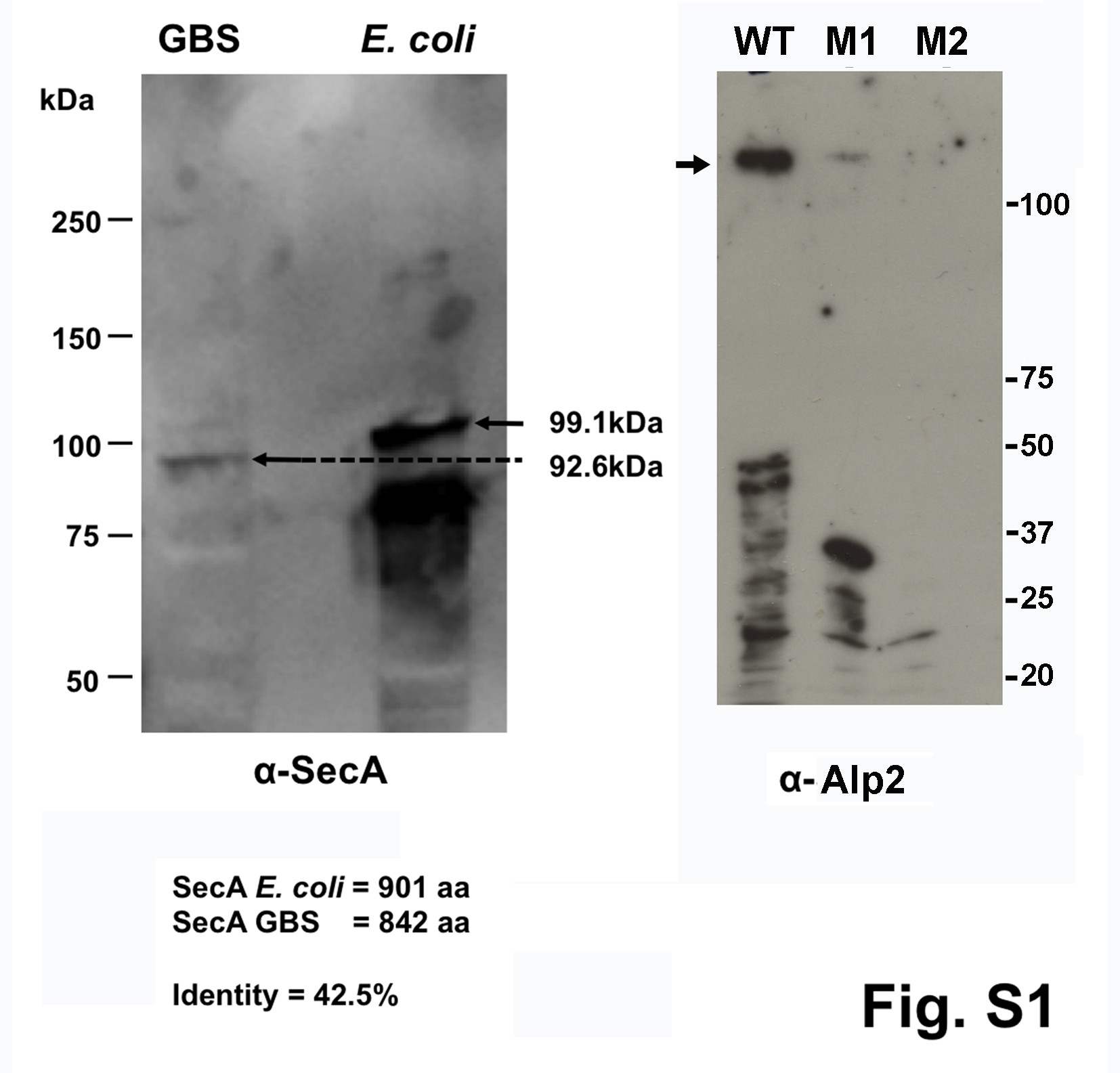

Supplement: Figure S1 — Western blotting showing that the polyclonal antibody directed against SecA of E. coli recognizes a band of approximately 90 kDa in GBS despite a low level of identity at the amino acid level. Western blot showing the specificity of Alp2 antiserum on the cell wall extracts from WT NEM316 and two transposon mutants inserted at two independent sites in gbs0470 (alp2) (M1: NEM3695 and M2: NEM3694). (TIF) [file pone.0065832.s001.tif]

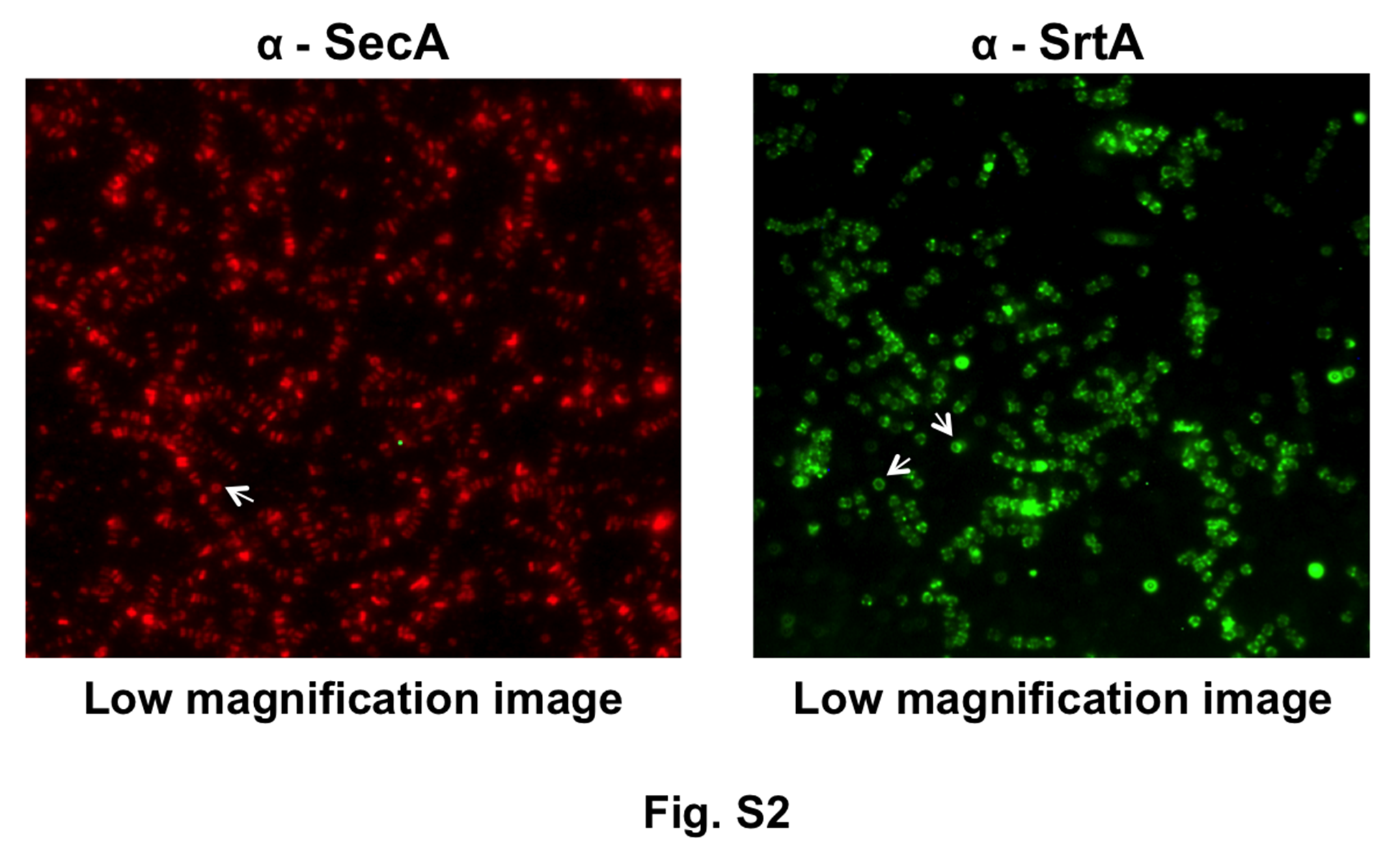

Supplement: Figure S2 — Low magnification image of SecA and SrtA labeling in GBS strain NEM316. Single cocci displaying a uniform labeling are indicated with white arrows. (TIF) [file pone.0065832.s002.tif]

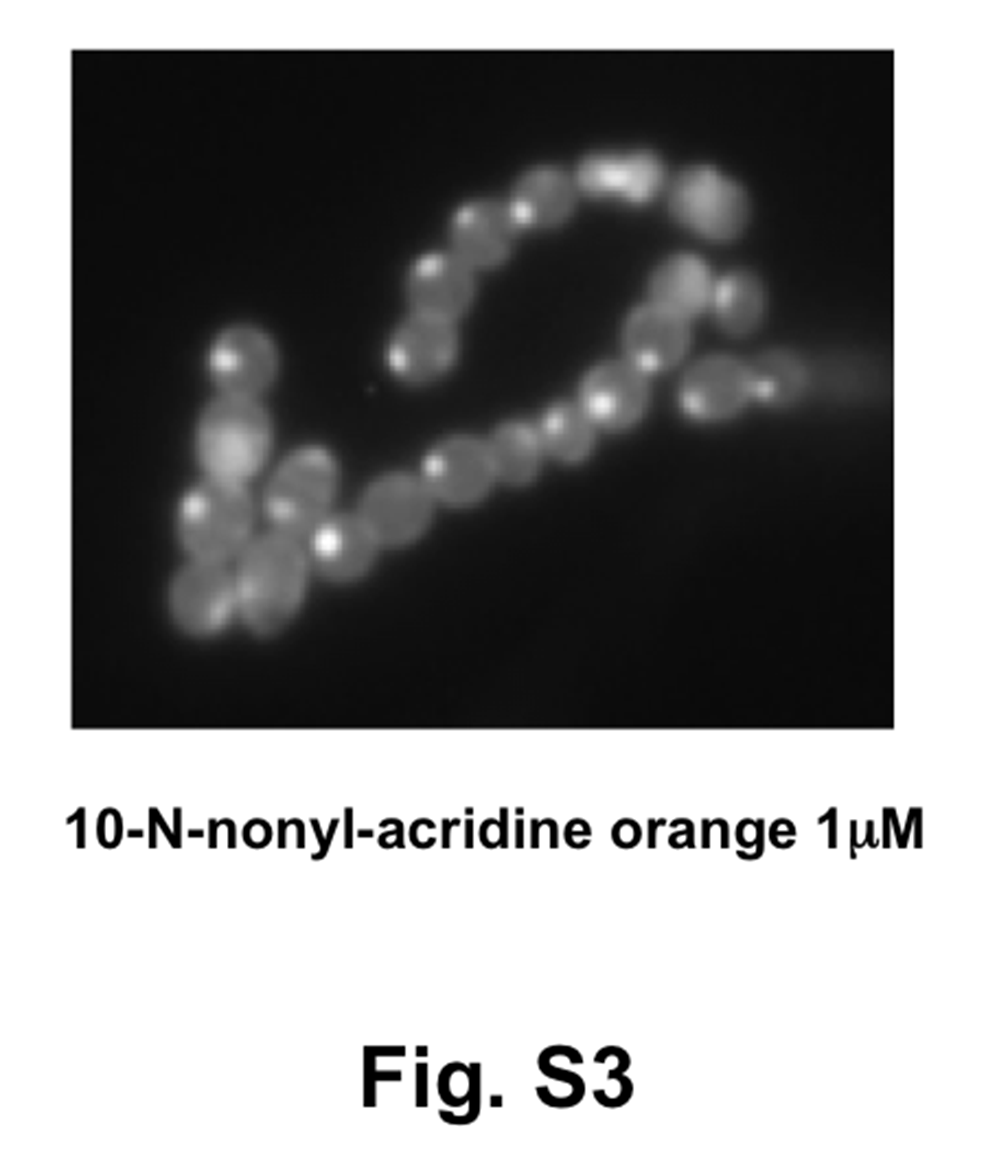

Supplement: Figure S3 — An ExPortal-like domain enriched in anionic lipids can be visualized using the NAO (10-N-nonyl-acridine orange 1 µM) dye in GBS strain NEM316. (TIF) [file pone.0065832.s003.tif]
