# Supplementary material for: SecA Localization and SecA-Dependent Secretion Occurs at New Division Septa in Group B Streptococcus
Source: PLoS One. 2013 Jun 7;8(6):e65832. doi: 10.1371/journal.pone.0065832 (PMC3676364; doi:10.1371/journal.pone.0065832)
Supplement: Table S1 — List of oligonucleotides used in this study. (DOCX) [file pone.0065832.s004.docx]

**Table S1. Oligonucleotides used in this study**

| **Bsp translational fusions** | |  |
| --- | --- | --- |
| bsp_NotIFwd | CATCGGTTCAAGTGCGGCCGCAGACAGGCACTAGTGTGGATGC |  |
| bsp_Pst | ATAATCTGCAGTTGACGTAAATCTCCACTGT |  |
| bsp_Bam | GATATTGGATCCGTAGTGTAAATAAATAAGGAAGTAGG |  |
| bsp_NotIRev | CTAGTGCCTGTCTGCGGCCGCACTTGAACCGATGTAGTTTGAT |  |
| pilB_Bam | TTATTGGATCCGTTGATTAACTAGAATAAGAAGGAG |  |
| pilB_NotIRev | CTTGTGCGGCCGCACAGTATCAGTTGTTACGTCGTCCGC |  |
| gbs0791_Bam | TAATTGGATCCGAAATATATTTTTTCTGGAGGAAAATAG |  |
| gbs0791_NotIRev | ACCTTGCGGCCGCCTTGCTGCCATTACTGGTGTC |  |
| alp2_Bam (alp2) | TAATTGGATCCGTATAGAGTTATACAGAGTAAAGGAG |  |
| alp2_NotIRev2 | TGTATGCGGCCGCGCTGCACTCCCTGGAATTGTAGA |  |
| cspA_Bam | TAATAGGATCCGTAATTATTATGTTACAAGAAAAGGAGA |  |
| cspA_NotIRev | AACTGGCGGCCGCGATGGCTTATTTATGACAGAATCAG |  |
| **___________________________________________________________________________________________**  **Histidine tagged recombinant fusion proteins** | |  |
| cpsA_NdeI (gbs2008) AATTCCATATGGATTCTGTCATAAATAAGCCATCT | | AATTCCATATGGATTCTGTCATAAATAAGCCATCT |
| cpsA_Bam (gbs2008) | AAAGGATCCCCCACTCATCATATCATATTCAT |  |
| gbs0791_NcoI | CATGCCATGGAGACACCAGTAATGGCAGCA |  |
| gbs0791_Bam | CGCGGATCCGGCGAACCTTGCTTTGCCATC |  |
| srtA_NcoI | CAATCCATGGATTATCAAATTTCACGAGTTAG |  |
| srtA_NotI | TTTCGGCGGCCGCTTTTAATATCGACTCATCTGCT |  |

The restriction sites used for cloning are underlined
